# Knowledge, Attitudes, and Practices Regarding Intravenous Fluids Among Physicians in the West Bank of Palestine: A Cross‐Sectional Study

**DOI:** 10.1002/hsr2.73148

**Published:** 2026-09-02

**Authors:** Mamoun Swaileh, Razan Rabi, Dina Abugaber, Yusuf B. Dawabsheh, Murad A. M. Karajeh, Halla Albada, Ziad Radad, Sa'ed H. Zyoud, Moutaz W. Sweileh

**Affiliations:** ^1^ Department of Internal Medicine An‐Najah National University Hospital Nablus Palestine; ^2^ Department of Medicine, Faculty of Medicine and Allied Medical Sciences An‐Najah National University Nablus Palestine; ^3^ Faculty of Medicine Al‐Quds University Jerusalem Palestine; ^4^ Department of Nursing Istishari Arab Hospital Ramallah Palestine

**Keywords:** attitudes, intravenous fluids, knowledge, Palestine, physicians, practices

## Abstract

**Background and Aims:**

Inappropriate use of intravenous (IV) fluids is associated with increased morbidity. Despite their routine use in hospitalized patients, physicians' knowledge, attitudes, and practices (KAP) regarding IV fluids remain inadequately investigated. This study assessed physicians' KAP in the West Bank, Palestine.

**Methods:**

A cross‐sectional online survey targeted physicians caring for hospitalized adult patients in West Bank hospitals. A questionnaire assessed demographic and professional characteristics, knowledge, attitudes, practices, and perceived barriers regarding IV fluids. Knowledge scores were converted into percentages and summarized using medians/interquartile ranges (IQR). Comparison of knowledge scores across physician subgroups was performed using the Mann–Whitney *U* test and the Kruskal–Wallis test, with Dunn's post hoc test and a Holm adjustment where appropriate. Attitudes, practices, and perceived barriers were presented as frequencies and percentages.

**Results:**

Of 256 participants (median age = 28 years, IQR 25–33), there were 173 (67.6%) males and 72 (28.1%) reported receiving formal IV fluids training during the previous 2 years. Residents and fellows comprised 101 (39.5%). The median knowledge score was 57.7% (IQR 35.7). The highest correct response in the knowledge section (*n* = 223, 87.1%) was observed for identifying hyperchloremic metabolic acidosis as a complication of 0.9% saline, while the lowest correct response (*n* = 67, 26.2%) was observed for identifying IV fluids not recommended for routine use. Knowledge scores differed significantly by departmental affiliation (*P* < 0.001) and professional position status (*P* = 0.03). Regarding attitudes, 116 (45%) agreed/strongly agreed that balanced crystalloids should be preferred over 0.9% saline in most ICU patients. In practice, 140 (54.7%) reported using 0.9% saline as the first‐line fluid in their practice. The most cited barrier was the absence of institutional guidelines (*n* = 157, 61.3%).

**Conclusion:**

Physicians demonstrated average knowledge, suboptimal practices, and limited agreement with evidence‐based attitudes toward IV fluid therapy. Educational interventions and hospital protocols are needed to help improve KAP.

## 1 Background

Intravenous (IV) fluids are integral to patients' therapeutic management plans. Inappropriate prescriptions of IV fluids have been associated with increased morbidity and worse outcomes [1]. Physicians responsible for prescribing and monitoring patients receiving IV fluids must understand fluid physiology, potential complications, and effects [2]. IV fluids have long been perceived as a harmless practice, and their administration is often guided by traditional habits rather than evidence‐based practices [3]. However, physicians should be aware of both the indications and potential adverse effects of various fluids [4, 5]. Previously published reports raised concerns about physicians' knowledge of IV fluids across different healthcare settings [6, 7, 8, 9, 10]. The reports revealed limited knowledge about IV fluids and different prescribing patterns and attitudes, leading to potential non‐uniform management plans regarding IV fluids.

The West Bank of Palestine is a small area located in the Middle East and is managed by the Palestinian National Authority [11, 12, 13]. In the West Bank of Palestine, there are three major healthcare providers for inpatient care: governmental hospitals, private hospitals, and teaching (university‐affiliated) hospitals. There is limited national or formal continuing medical education for physicians in the West Bank of Palestine to keep them updated. Furthermore, healthcare providers are overwhelmed by high patient volumes, and hospitals have limited financial resources, which adds further challenges for healthcare providers to remain medically updated. Although IV fluids are a critical part of the care of hospitalized or critically ill patients, there is limited information in the West Bank of Palestine regarding physicians' knowledge, attitudes, and practices (KAP) related to IV fluids.

To the best of our knowledge, this is the first physician‐focused study addressing adult inpatient KAP in the West Bank and among the Arab countries. Despite the clinical importance of IV fluids, studies from Arab countries remain limited and primarily focused on selected aspects rather than the overall physicians' KAP. Previous reports from the region have examined topics such as volume assessment practices [14], pediatric IV fluids knowledge and practices [15], implementation of National Institute for Health and Care Excellence (NICE) IV fluids guidelines in postoperative patients [16], and nurses' KAP studies [17, 18].

Therefore, the primary objective of the current study was to assess physicians' KAP regarding IV fluids across hospitals in the West Bank of Palestine. Secondary exploratory objectives were to examine variations across physicians' groups and identify areas requiring targeted educational interventions.

## 2 Methods

### 2.1 Study Design

This was a cross‐sectional online survey conducted to assess physicians' KAP regarding IV fluids. The study was conducted in accordance with the “Strengthening the Reporting of Observational Studies in Epidemiology” (STROBE) guidelines. The STROBE checklist is included as Supplement 1.

### 2.2 Study Settings and Period

The current study was carried out in hospital settings across governmental, private, and teaching hospitals in the West Bank of Palestine. The study was conducted from November 01, 2025 to January 31, 2026.

### 2.3 Participants and Recruitment

Eligible participants included physicians, regardless of their professional status (interns, residents, fellows, specialists, and consultants), currently practicing in West Bank hospitals. The survey was distributed online through hospital email lists, department and professional WhatsApp groups, and related social media forums. Participation was voluntary and anonymous. Electronic informed consent was required before accessing the questionnaire. Each participant was asked to complete the survey once. Professional roles were self‐reported by the participants; no external verification was performed. For clarity, interns are first‐year medical graduates who rotate across different departments in supervised roles. Residents are physicians in postgraduate specialty training. Fellows are physicians undertaking subspecialty training after residency. Specialists are attending physicians who have completed specialty training and practice independently, while consultants are senior attending physicians with extensive experience and supervisory or leadership roles. Pediatric and outpatient settings were not included because IV fluid prescribing practices, indications, and monitoring differ from adult patients. They follow different guidelines and principles. Outpatient settings were also excluded because IV fluid use is more limited and occurs in a different clinical context compared to inpatient resuscitation and maintenance IV fluids. Therefore, the study focused on adult inpatient settings for a more homogeneous assessment.

### 2.4 Questionnaire Development

The questionnaire was developed for this study by three authors with relevant experience related to IV fluids. Item generation was informed by international guidelines, including NICE guidelines [19], Surviving Sepsis Campaign [20], and recommendations from the International Fluid Academy [1]. Item generation was also informed by relevant published literature and previous surveys of physicians' knowledge and practices regarding IV fluids [21, 22, 23, 24]. The multidomain structure was informed by those prior surveys, which commonly assessed combinations of physicians' characteristics, knowledge, prescribing practices, and perceptions regarding IV fluids. Consistent with these surveys' questionnaire tools, the present study incorporated core domains of KAP. This study expanded on previous work by including prior formal training and awareness of institutional guidelines to provide a broader assessment. The questionnaire is attached as Supplement 2.

### 2.5 Survey Sections

The final questionnaire included five sections: demographics, knowledge, attitudes, practices, and barriers. The demographic and professional characteristics section included age, gender, departmental affiliation, current professional position, hospital type, prior formal training regarding IV fluids in the past 2 years, and the presence of institutional IV fluids guidelines/protocols. The knowledge section comprised 12 scored items (11 single‐best‐answer items; item 12 contained three correct options), yielding a raw score of 0–14. They were designed to assess clinically relevant aspects of IV therapy, including maintenance fluids requirements, resuscitation fluids, electrolyte considerations, and fluid responsiveness assessment. Each correct option scored one point. Incorrect or ‘I don't know' responses scored zero. The scores were then summed, converted into a percentage, and analyzed as a continuous variable. The knowledge score was categorized into five levels for interpretation: ≥ 80% (excellent), 60–79% (above average), 40–59% (average), 20‐39% (below average), and < 20% (poor) [22]. Data on prior formal training and hospital protocols were collected as an exploratory variable to explore potential associations with physicians' knowledge or practice.

Attitude items assessed preferences for balanced crystalloids versus 0.9% saline, the importance of education, audit, and stewardship strategies in optimizing IV fluids therapy. Items were measured using six 5‐point Likert items, ranging from strongly disagree to strongly agree. Attitude items are reported descriptively as frequencies and percentages of respondents in each response category. In the current study, combined responses of “strongly agree” and “agree” greater than 80% were considered to indicate high agreement, whereas values below 60% indicated limited agreement. Percentages between 60% and 80% were classified as intermediate agreement [25]. Practices were assessed with seven self‐reported items assessing real‐world prescribing patterns and documentation behavior. Barrier items were predefined responses derived from commonly reported obstacles in prior reports [21, 24]. Practice responses and cited barriers were presented as frequencies and percentages for each item.

The survey was hosted on Google Forms, and all questionnaire items were mandatory, ensuring no missing data. Because the questionnaire required completion of all items before submission in Google Forms, partially completed questionnaires were not recorded. Responses were exported to a secure and protected database. The questionnaire was administered in English. Although Arabic is the native language in the West Bank of Palestine, English is the official language of medical education, clinical documentation, electronic medical records, and licensing examinations. Consequently, physicians in the West Bank routinely use English in daily clinical practice and academic activities. Therefore, the use of an English‐based questionnaire was considered appropriate, and no major language‐related comprehension difficulties were anticipated.

### 2.6 Sampling Bias

To reduce selection bias, the survey was distributed across multiple professional platforms and hospital networks, targeting physicians from various specialties and hospital sectors. Participation was voluntary and anonymous, which may have reduced social desirability bias and encouraged honest responses. However, as the data were self‐reported, the possibility of recall and reporting bias cannot be entirely excluded.

### 2.7 Study Size

The sample size was estimated to ensure adequate precision for descriptive estimates of physicians' KAP regarding IV fluids. In the absence of prior local data, a 50% prevalence of adequate knowledge was assumed to maximize the required sample size. Using the Raosoft calculator, the target sample size was 257 respondents at a 90% confidence level and a 5% margin of error [26]. A 90% confidence level was used to estimate a feasible sample size for this descriptive study. A total of 256 physicians completed the survey during the study period. Due to the distribution across multiple open channels, the total number of physicians was not predetermined. Subgroup comparisons of knowledge scores across physician characteristics were predefined as exploratory analyses and were not used as the basis for sample size estimation. As the number of physicians who received the survey is unknown, the response rate could not be calculated.

### 2.8 Statistical Analysis

Data analyses were performed using IBM SPSS Statistics software version 31 (IBM Corp, Armonk, NY, USA). The normality of the knowledge score distributions was assessed using the Kolmogorov–Smirnov test and was found to be non‐normal. Knowledge scores were treated as continuous variables and summarized using medians and interquartile ranges (IQR). Categorical variables, including attitudes, practices, and barriers, were presented as frequencies and percentages. As the study was primarily descriptive, no formal power calculation was performed for subgroup comparisons, which were considered exploratory in nature. Comparison of knowledge scores between two groups was performed using Mann–Whitney U tests, while comparisons across more than two groups were conducted using the Kruskal–Wallis test. Where statistically significant differences were identified, post hoc pairwise comparisons were performed using Dunn's test with Holm adjustment to account for multiple testing. All statistical tests were two‐sided. A *p* value < 0.05 was considered statistically significant. Regarding values, they were reported according to standard reporting conventions: values < 0.001 were reported as *p* < 0.001, values between 0.001 and 0.01 were reported to three decimal places, and values of 0.01 or more were reported to two decimal places.

### 2.9 Validity and Reliability of the Questionnaire

Content validity was ensured through a multistep process. The initial questionnaire was drafted by three of the authors, guided by international guidelines and reports. A preliminary version of the questionnaire underwent expert review by seven specialists and consultants with expertise in IV fluids, including critical care, emergency, and anesthesia departments. They evaluated the instrument for clinical relevance and clarity. Based on their feedback, two unclear items were removed, and two questions underwent minor refinement. Thereafter, a pilot study was carried out prior to the start of the survey, including 10 physicians from different specialties, and minor wording and formatting adjustments were made based on their feedback. The internal consistency of the attitude section (the Likert scale items) was evaluated via Cronbach's alpha (*α* = 0.86), which showed acceptable reliability. For the knowledge and practice sections, reliability was not examined, as this is a characteristic of sections that are considered formative, guideline‐referenced, and behavior‐oriented in nature, where each item is considered a unique and non‐substitutable part of practice and cannot be considered an indicator of a singular construct; hence, Cronbach's alpha is not an appropriate measure for use in this case.

### 2.10 Ethical Considerations

All the participants provided electronic informed consent prior to survey completion. No patient‐level data were collected. The study was conducted in accordance with ethical principles and was approved by the Institutional Review Board (IRB) of An‐Najah National University (approval number: Pharm. Oct. 2025/141).

## 3 Results

### 3.1 Descriptive Characteristics of Participants

A total of 256 physicians answered the questionnaire and were included in the analysis. The median age of the participants was 28 years (IQR = 25–33). There were 173 (67.6%) male participants. Regarding departmental affiliation, the majority (*n* = 69, 27.0%) were affiliated with the internal medicine department. Regarding the professional position, the majority (*n* = 101, 39.5%) were residents and fellows, followed by interns (*n* = 89, 34.8%). Regarding hospital affiliation, 132 (51.5%) were affiliated with governmental hospitals, 70 (27.3%) with teaching hospitals, and 54 (21.1%) with private hospitals. Seventy‐two (28.1%) of the participants reported that they received formal IV fluids training in the past 2 years, and 73 (28.5%) reported the presence of institutional guidelines for IV fluids (Table 1).

**Table 1:** Demographic and professional characteristics of participating physicians.

Characteristics	Statistics
Age	Median (IQR) = 28 (25–33)
Gender	*n* (percentage of participants)
Male	173 (67.6%)
Female	83 (32.4%)
Department*	*n* (percentage of participants)
Critical care	6 (2.3%)
Anesthesiology	18 (7.0%)
Emergency medicine	32 (12.5%)
Internal medicine	69 (27.0%)
Surgery	23 (9.0%)
Others (neurology, orthopedics, neurosurgery, etc.)	19 (7.4%)
Current position	*n* (percentage of participants)
Residents and Fellows	101 (39.5%)
Intern	89 (34.8%)
Specialist	51 (19.9%)
Consultant	15 (5.9%)
Hospital type	*n* (percentage of participants)
Governmental	132 (51.6%)
Private	54 (21.1%)
Teaching (University‐affiliated)	70 (27.3%)
Formal IV fluids training within the last two years	*n* (percentage of participants)
Yes	72 (28.1%)
No	184 (71.9%)
Presence of local IV fluids guidelines	*n* (percentage of participants)
Yes	73 (28.5%)
Not sure	89 (34.8%)
No	94 (36.7%)

Abbreviations: IQR, interquartile range; IV, intravenous.

*Interns were not calculated as part of any specific department.

### 3.2 Knowledge

Knowledge item performance is shown in Table 2. High correct response rates were observed for basic fluid composition and safety items. For example, 223 (87.1%) of the participants identified *“hyperchloremic metabolic acidosis as a complication of 0.9% saline solution”* and 203 (79.3%) identified “*the need for at least daily review of fluid prescriptions.”* Correct responses were lower for clinical decision‐making knowledge items. For example, 96 (37.5%) of the participants correctly selected “*balanced crystalloids as the first‐line resuscitation fluid in sepsis patients,”* and 67 (26.2%) correctly identified “*IV fluid types not recommended for routine use*.” The median (IQR) knowledge score was 57.7% (IQR = 35.7). Knowledge scores varied significantly across departmental affiliations (*p* < 0.001) and current professional positions (*p* = 0.03). Median knowledge was highest for participants affiliated with the internal medicine department (71.0%, IQR 36) and lowest for participants affiliated with the surgery department (50.0%, IQR 14.3). Post‐hoc analysis showed significant differences between internal medicine and surgery (*p* = 0.01), with participants in internal medicine having significantly higher knowledge scores than those in surgery. No statistically significant differences in knowledge scores were observed based on gender (*p* = 0.94), age (*p* = 0.61), hospital type (*p* = 0.28), or previous formal IV fluids training in the past 2 years (*p* = 0.24). The findings showed an overall knowledge score of 57.7%, which falls within the average predefined category. Comparison of knowledge scores by physician characteristics is shown in Table 4.

**Table 2:** Knowledge items performance regarding intravenous fluids.

	Knowledge domain	Correct answer	Number of participants answered correctly (percentage of participants)
1	Daily maintenance requirement	Water 25–30 mL/kg; Na 1 mmol/kg; K 1 mmol/kg; Glucose 50–100 g	170 (66.4%)
2	Solution closest to plasma SID	Balanced crystalloids (LR/Plasmalyte)	188 (73.4%)
3	Main complication of 0.9% saline	Hyperchloremic metabolic acidosis	223 (87.1%)
4	Sepsis resuscitation	30 mL/kg	131 (51.2%)
5	First‐line resuscitation fluid	Balanced crystalloids	96 (37.5%)
6	Reasonable bolus size	250–500 mL	119 (46.5%)
7	Best test of fluid responsiveness	PLR with CO/SV	118 (46.1%)
8	Balanced fluids in CKD	Usually safe with monitoring	155 (60.5%)
9	IV fluids not recommended for routine use	Hypertonic saline	67 (26.2%)
10	Maintenance rate (70 kg adult)	1–1.5 mL/kg/h	148 (57.8%)
11	Minimum review frequency	At least daily	203 (79.3%)
12	Acceptable dynamic assessments for fluid responsiveness	Number of correct options selected	
		Passive leg raise with CO/SV change	1 correct: 92 (36.0%)
		Echocardiographic VTI/IVC variation	2 correct: 74 (29.0%)
		Pulse pressure variation	3 correct: 70 (27.0%)

Abbreviations: CKD, chronic kidney disease; CO, cardiac output; ICU, intensive care unit; IVC, inferior vena cava; PLR, passive leg raise; SID, strong ion difference; SV, stroke volume; and VTI, velocity time integral.

### 3.3 Attitudes

**Figure 1:**
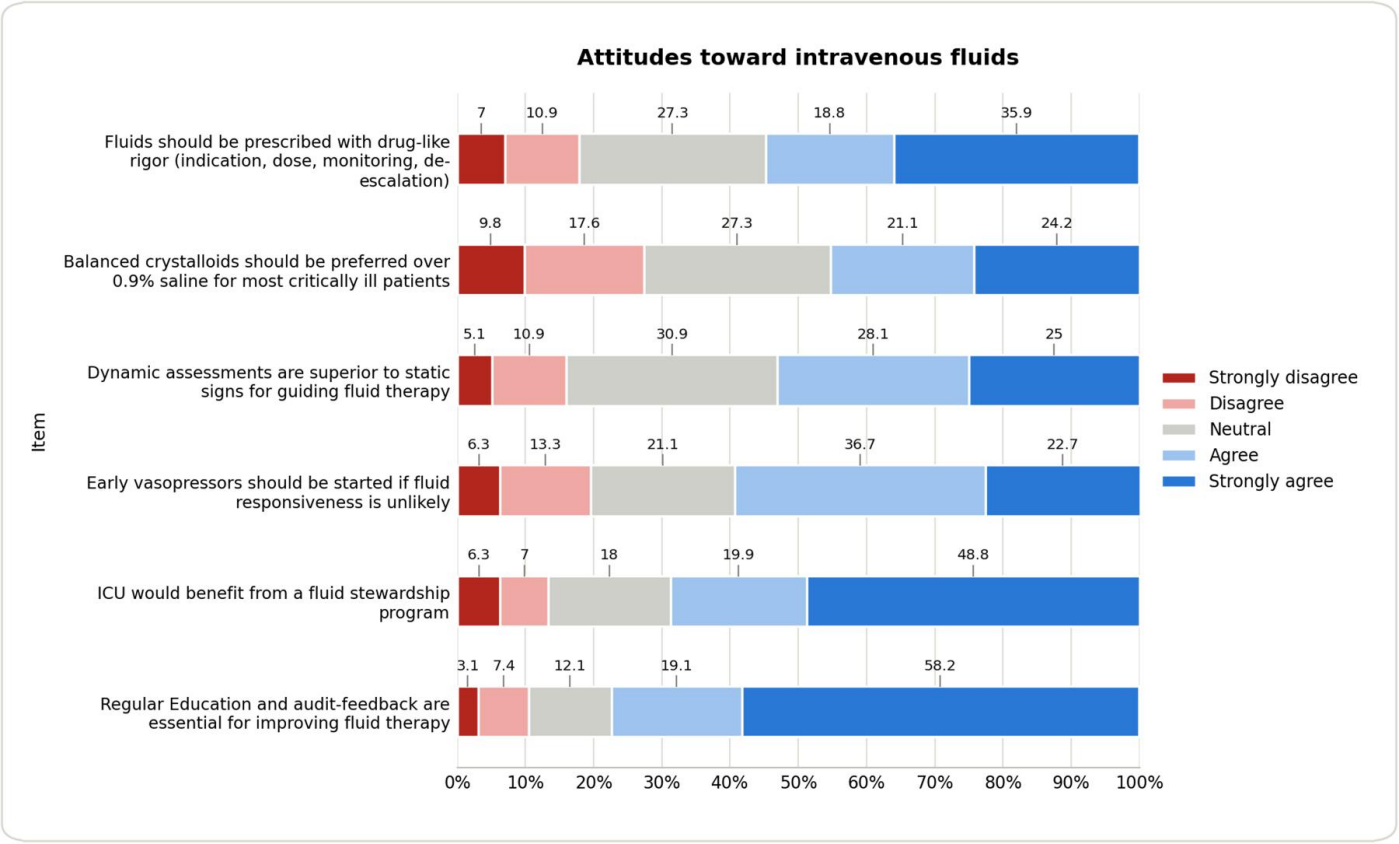
Physicians' attitudes toward intravenous fluids.

Regarding participants' attitudes, 198 (77.0%) agreed/strongly agreed that *“Regular education and audit feedback are essential for improving fluid therapy”* and 116 (45.0%) of the participants agreed/strongly agreed that “Balanced *crystalloids should be preferred over 0.9% saline for most critically ill patients.”* The remaining items in the attitude domain are listed in Figure 1. The overall proportion of agreement across attitude items was 59.7%, which corresponds to limited agreement according to the predefined criteria, although it is close to the cutoff for intermediate agreement.

### 3.4 Practices

Practice items are reported as frequencies and percentages in Table 3. For example, 140 (54.7%) reported using “0.9% s*aline”* as their most commonly used first‐line resuscitation fluid, 25 (9.8%) reported using “Albumin” as their first‐line resuscitation fluid, 186 (72.7%) reported using *“500 mL over 10–15 min”* as their typical bolus administration volume in an unstable adult patient, while 199 (77.7%) reported “Reassessing Fluids every 4–6 h.” Regarding documentation practices, 227 (88.7%) reported that they “*Routinely recorded fluid type, dose, and rate,”* while 121 (47.0%) documented *“Indication.”*.

**Table 3:** Self‐reported practices regarding intravenous fluids among participating physicians.

Practice item	Number (percentage of participants)
First‐line resuscitation fluid used in the ICU	
Balanced crystalloids	91 (35.5%)
0.9% saline	140 (54.7%)
Albumin	25 (9.8%)
Usual bolus volume in an unstable adult patient	
500 mL over 10–15 min	186 (72.7%)
250 mL over 10–15 min	27 (10.5%)
500 mL over 30–60 min	21 (8.2%)
Other	22 (8.6%)
Frequency of Re‐evaluation of IV Fluids	
Every 4–6 h	199 (77.7%)
Every 48 h	47 (18.4%)
Every 72 h	6 (2.3%)
Weekly	4 (1.6%)
Routine documentation includes *	
Indication	121 (47.0%)
Fluid type, dose, rate	227 (88.7%)
Cumulative fluid balance	137 (53.0%)
Weight	113 (44.0%)
None	4 (1.6%)
Does Chloride Monitoring Influence Your Fluid Choice	
Always	53 (20.7%)
Often	95 (37.1%)
Sometimes	71 (27.7%)
Rarely	37 (14.5%)
Avoidance of HES/Gelatin Solutions	
Always	91 (35.5%)
Often	98 (38.3%)
Sometimes	49 (19.1%)
Rarely	18 (7.0%)
Use of balanced crystalloids for drug dilution when compatible	
Yes	166 (64.8%)
No	27 (10.5%)
Not sure	63 (24.6%)

* Multiple options allowed

Abbreviations: HES, hydroxyethyl starch; ICU, intensive care unit.

**Table 4:** Comparison of knowledge scores by physician characteristics.

Characteristics	Knowledge Score (%), Median (IQR)	*p* value
Gender		*p* = 0.94
Male	57.0% (35.7)	
Female	57.1% (28.0)	
Age group (Years)		*p* = 0.61
< 30	57.1% (39.0)	
30–39	57.0% (35.0)	
≥ 40	57.0% (42.0)	
Specialty		*p* < 0.001
Internal medicine	71.0% (36.0)	
Anesthesiology	67.0% (32.0)	
Critical care	78.5% (28.5)	
Surgery	50.0% (14.3)	
Emergency medicine	57.0% (41.1)	
Others	50.0% (33.9)	
Current position		*p* = 0.03
Consultant	71.0% (35.7)	
Intern	50.0% (35.7)	
Resident and Fellow	57.0% (28.6)	
Specialist	64.0% (43.0)	
Previous formal training		*p* = 0.24
Yes	57.1% (35.7)	
No	56.5% (42.8)	
Hospital type		*p* = 0.25
Governmental	53.0% (36.0)	
Private	57.0% (35.0)	
Teaching (University‐affiliated)	57.0% (35.0)	
Overall median knowledge score	57.7% (35.7)	

Abbreviation: IQR, Interquartile Range.

Knowledge scores presented as median (IQR) percentages.

Comparisons between two groups were performed using the Mann–Whitney *U* test, whereas comparisons across more than two groups were performed using Kruskal–Wallis tests.

### 3.5 Barriers

**Figure 2:**
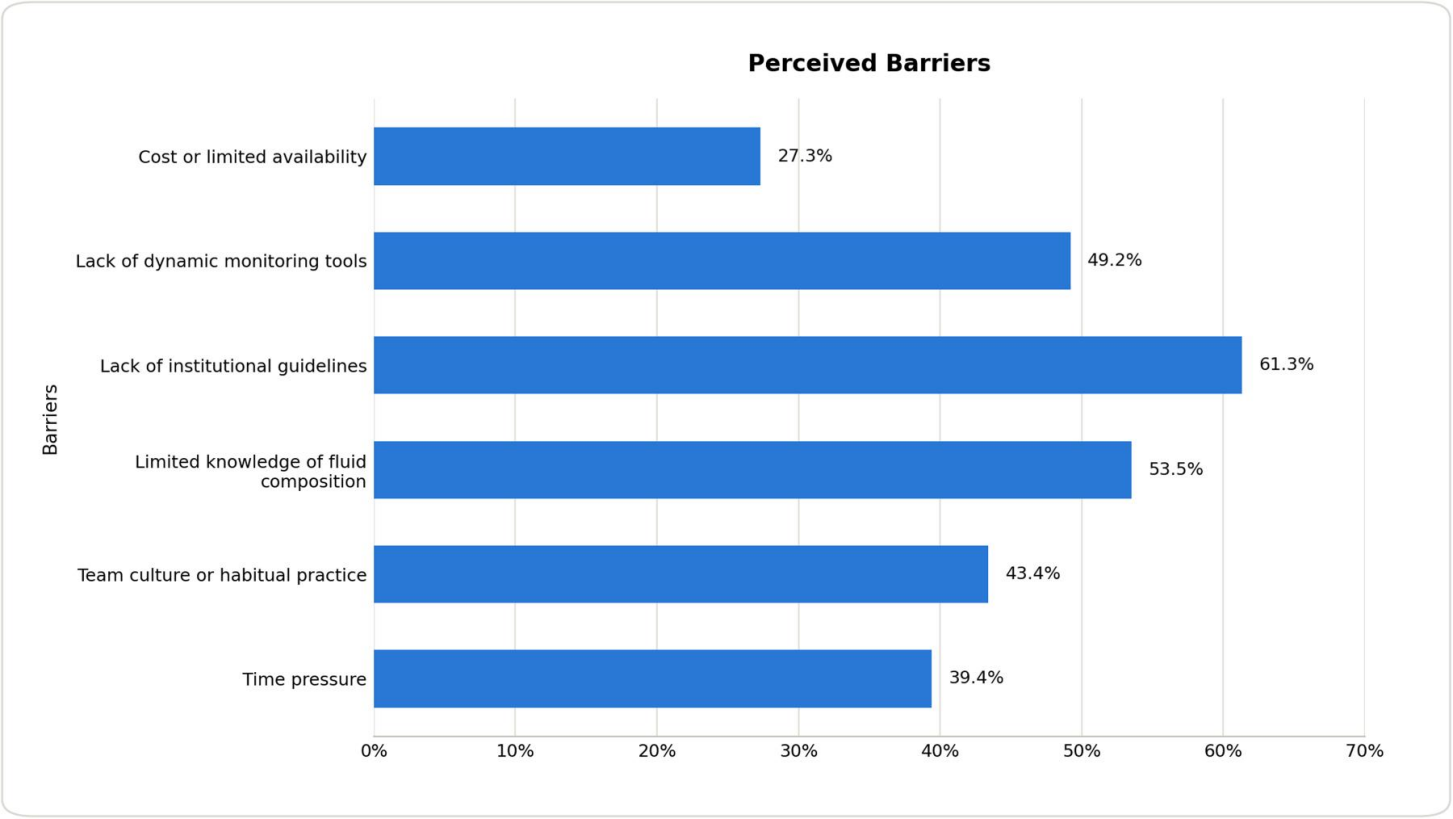
Perceived barriers to optimal intravenous fluids prescribing.

The participants identified multiple barriers to optimal IV fluids management (Figure 2). One hundred and fifty‐seven (61.3%) of the participants reported that the “*absence of institutional guidelines*” was a barrier, while 70 (27.2%) of the participants reported “*cost*” as a barrier.

## 4 Discussion

The current study evaluated physicians' KAP regarding IV fluids in the West Bank of Palestine. The findings showed an average knowledge score, limited agreement on implementing items to improve the use of IV fluids, and suboptimal fluid prescribing practices. The major reported barrier for physicians to achieving optimal use was the absence of institutional guidelines.

The current study showed a discrepancy between knowledge and practice. For example, although a high percentage of participants acknowledged hyperchloremic metabolic acidosis from 0.9% saline, a relatively low percentage reported using balanced crystalloids as the first‐line resuscitation fluid. This discrepancy has been reported in other countries [23]. Lower performance for certain items in the knowledge section mirrors findings from international surveys. For example, only 43.0% of physicians in a Belgian survey used the dynamic assessment method (passive leg raise test) to assess fluid responsiveness, which is close to the score reported in the current study [24]. A multicenter global survey showed that physicians more commonly use static rather than dynamic IV fluids assessment tools [22]. These results indicate that the knowledge‐practice gap is widespread and not confined to low‐income countries. The findings in this study also showed a knowledge deficit regarding maintenance fluids, which is consistent with a previously published study among junior physicians revealing widespread uncertainty in this domain [27]. A published scoping review showed that junior physicians are underprepared and must have a clearer understanding of IV fluids [28]. Knowledge scores differed significantly across departmental affiliations, with greater knowledge scores among physicians in critical care, internal medicine, and anesthesiology. This could be explained by the fact that those physicians have relatively greater exposure to IV fluid therapy compared with physicians in other departments. These results are consistent with a previous survey indicating that physicians in critical care departments have better knowledge of IV fluids than their colleagues in non‐critical fields, likely because of greater exposure, training, and frequent clinical application [21, 22]. Consultants scored significantly higher on knowledge scores than interns. While greater knowledge is expected among consultants or specialists, given their experience, the interns and junior residents, who commonly prescribe initial IV fluids, had lower knowledge scores. Therefore, they should represent primary targets for educational intervention for future IV fluids training courses. Habitual practice patterns and institutional culture likely drive IV fluids selection. This indicates that physicians often prescribe IV fluids based on “shortcuts” learned during their careers rather than relying on evidence, investigations, and examinations, which may explain the current practice patterns [28]. System‐based interventions may, therefore, be required to translate knowledge into practice. Poor documentation of cumulative fluid balance, clinical indications, and patient weight may lead to excessive or inadequate IV fluids administration [28]. Structured charts and standardized documentation could improve IV fluids prescription practices.

The United Kingdom NICE guidelines provide comprehensive recommendations for IV fluids assessment, fluid choice, dosing, review frequency, and documentation for adult patients [19]. These national standards can inform local practices and local hospital protocols. However, experience from London and Scotland showed that, despite the availability of IV fluids guidelines, knowledge of, and compliance with, those guidelines remain suboptimal [27]. Reasons for that are likely multifactorial. For example, limited awareness of, or easy access to, the guidance, a perception of IV fluids as a routine task unworthy of study, lack of local order sets/protocols, and workflow constraints. Therefore, alongside endorsing national guidelines, hospitals should translate them into locally usable tools (like ward algorithms for IV fluids or order sets).

Regarding attitudes, although most participants supported education and audit, the composite attitude score was classified as limited. It seems that habits and system constraints play a role in shaping physicians' attitudes toward IV fluids. Many physicians know that IV fluids do matter; however, the habit of viewing IV fluids as routine therapy may still exist. Practices in this survey showed a lag behind knowledge, reflecting habitual prescribing and inconsistent documentation across a few domains. Regarding barriers, the lack of institutional guidelines was the most reported one rather than costs. This suggests that the implementation of evidence‐based IV fluids practices in the West Bank is feasible if organizational support is provided. Notably, formal training in intravenous fluid therapy did not predict a statistically significantly higher knowledge score. This indicates potential deficiencies in the educational content and delivery of training programs.

### 4.1 Recommendations

Based on the identified gaps in knowledge and variability in prescribing behaviors, improvement efforts should extend beyond education to practical system‐level interventions. First, hospitals should develop local IV fluids protocols adapted from established guidance, for example NICE guidelines and the Surviving Sepsis Campaign [19, 20]. These protocols should address common inpatient scenarios like maintenance fluids type and volume, electrolyte follow‐up, fluid replacement, and resuscitation. To improve and facilitate their use, protocols should be converted into ward algorithms and pocket references. Second, structured training should target physicians who commonly initiate and prescribe fluids, particularly interns and residents, as they prescribe fluids the most with minimal training and expertise in that field. Training should include bedside teaching, simulation exercises, and competency assessments, as educational interventions have been associated with improvements in IV fluids practices [27, 28]. Training should emphasize IV fluids selection, volume, reassessment of response, and recognition of potential complications. Third, multidisciplinary strategies should be considered. A fluid stewardship approach, similar to antimicrobial stewardship, may help standardize and improve IV fluids prescribing patterns [5]. This approach usually includes collaboration among physicians, nurses, pharmacists, and quality improvement teams to ensure protocol adherence. Nurses and pharmacists' involvement is important because of their involvement in administration, monitoring, and documentation. Fourth, improving awareness should rely on routine institutional processes rather than individual initiative alone. Practical steps may include regular educational sessions, refresher training, incorporating IV fluids review into ward rounds, handover sheets, and providing periodic feedback to the team [29]. Finally, hospitals should improve documentation practices by making standardized fluids charts or electronic templates which include fluid type, indication, volume, cumulative fluid balance, and patient weight [30].

### 4.2 Strengths and Limitations

This study has several strengths. As far as we are aware, this is among the first physician‐focused studies evaluating KAP regarding adult hospitalized patients receiving IV fluids in the West Bank of Palestine and among the limited available reports from Arab settings. The inclusion of physicians from multiple specialties, hospital types, and professional positions enhances the breadth of perspectives and diversity of clinical contexts captured in this study. Additionally, the assessment of multiple domains regarding IV fluids knowledge, attitudes, practices, barriers, and demographic data makes the questionnaire well‐structured with a systematic assessment. A methodological strength is the systematic development of the questionnaire using international guidelines, published literature, and expert‐panel review, which enhanced its content validity and clinical relevance. A further strength is the complete dataset, with no missing responses due to mandatory questionnaire completion, ensuring analytical consistency across all variables.

Several limitations also exist, such as the cross‐sectional design, which precludes causal inferences between physician characteristics and knowledge or practice patterns. Additionally, the recruitment was conducted through online channels, so the response rate could not be calculated. The representativeness of the sample relative to the overall physician workforce in the West Bank hospitals cannot be fully confirmed due to the open and non‐probability sampling approach. In addition, nonresponse bias and selection bias are possible. The recall bias and social desirability bias also cannot be excluded. The practices were self‐reported rather than verified through chart review and observation, which means that reported practices may differ from the actual bedside behaviors. Some subgroup sizes were small, such as the underrepresentation of critical care physicians and consultants, limiting the precision. Subgroup comparisons were exploratory in nature and not adjusted for multiple comparisons; therefore, the observed differences should be interpreted cautiously. Finally, findings may not be generalizable beyond similar healthcare contexts.

## 5 Conclusions

Physicians demonstrated reasonable basic safety knowledge regarding IV fluids, but they had important clinically relevant gaps in IV fluids decision‐making and practices that differed by departmental affiliation and professional position. Addressing both physicians' education and system‐level interventions (protocols, monitoring access, and stewardship) may be warranted to standardize practice and reduce harm.

## Author Contributions

**Mamoun Swaileh:** writing – original draft, writing – review and editing. **Razan Rabi:** formal analysis, writing – original draft, data curation. **Dina Abugaber:** conceptualization, supervision, resources. **Yusuf B. Dawabsheh:** investigation, project administration, writing – original draft. **Murad A.M. Karajeh:** validation, visualization, project administration. **Halla Albada:** software, project administration, writing – original draft, conceptualization. **Ziad Radad:** conceptualization, writing – original draft, project administration. **Sa'ed H. Zyoud:** formal analysis, methodology, conceptualization, supervision. **Moutaz W. Sweileh:** writing – original draft, conceptualization, project administration, validation.

## Artificial Intelligence Use Disclosure

The authors used Claude (Anthropic), an artificial intelligence‐based language model, to assist with manuscript editing, figure regeneration, and formatting during the revision process in response to the reviewer and editor comments. Artificial intelligence was not used for study design, data collection, data analysis, interpretation of results, or to generate scientific content. All edits were reviewed and verified by the authors, who take full responsibility for the integrity and accuracy of the manuscript.

## Funding

The authors have nothing to report.

## Conflicts of Interest

The authors declare that they have no conflicts of interest related to this study.

## Transparency Statement

The corresponding author, Dr. Dina Abugaber, affirms that this manuscript is an accurate, honest, and transparent account of the study being reported, that no important aspects of the study have been omitted, and that any discrepancies from the study as planned have been explained.

## Supporting information


Supporting File 1

## Data Availability

The data supporting the findings of this study are available from the corresponding author on reasonable request.
